# Artificial intelligence in drug discovery: from algorithmic foundations to clinical translation

**DOI:** 10.3389/fphar.2026.1870527

**Published:** 2026-07-03

**Authors:** Zihan Mao, Lihui Yan

**Affiliations:** 1 Department of Colorectal Surgery, Cancer Hospital of Dalian University of Technology, Liaoning Cancer Hospital & Institute, Shenyang, China; 2 Department of Pain Relief Therapeutic, Cancer Hospital of Dalian University of Technology, Liaoning Cancer Hospital & Institute, Shenyang, China

**Keywords:** artificial intelligence, drug discovery, machine learning, protein structure prediction, target identification

## Abstract

Artificial intelligence is quietly reconfiguring the landscape of drug discovery. It sharpens the search for new targets, refines lead compounds, and helps tailor clinical trial designs. Behind these advances sits a family of methods—convolutional, recurrent, and graph neural networks, generative adversarial networks, variational autoencoders, diffusion models, and Transformers—that has found its way into every stage of the pipeline. Tasks that once demanded years of trial and error, from pulling meaningful features out of molecules to predicting drug-target affinity and crystal structures with polymorph stability, now run faster and often with greater accuracy. This early insight lets researchers flag solid-form properties that influence bioavailability and manufacturability, trimming timelines and, in principle, lifting success rates. To map a field that keeps shifting shape, we searched PubMed, Web of Science, Scopus, and Google Scholar for peer-reviewed reports published between January 2007 and April 2026. The story the clinical cases tell is double-edged: one AI-discovered candidate has reached Phase IIa with encouraging efficacy, another stalled in Phase I when safety signals surfaced. AI can catch adverse effects earlier in toxicity assessments, yet nagging hurdles endure—biological complexity, patchy data, and a shortage of scientists fluent in both machine learning and pharmaceutics. Digging ourselves out will demand data standardization, nimble regulatory frameworks, and cross-disciplinary training. Weaving multimodal data together with explainable AI is becoming non-negotiable for transparency and regulatory confidence. The technology is now stretching into complex systems like Traditional Chinese Medicine and natural product screening. As AI continues to mature, it will reshape drug development in ways we are only beginning to grasp, but one truth remains stubborn: clinical translation still rests on rigorous experimental evidence.

## 1 Introduction

Developing a drug that is both effective and safe has always been a slow, expensive gamble. The meter runs faster at each milestone, making it critical to pick the right candidates early—especially during lead discovery and validation (Tr et al., 2023; Keserű and Makara, 2009). Roughly 90% of molecules that enter the clinic never make it out. In Phase II, 50%–60% of those failures trace back to a simple problem: the drug did not work, while another 30% come from unacceptable safety signals (Sun et al., 2022). Polypharmacology sits at the heart of many of these disappointments—a drug’s tendency to brush against multiple targets rather than just the intended one (Hopkins, 2008). Sometimes that breadth is therapeutic, but it can just as easily trigger side effects through tangled pathways that are hard to untangle prospectively (Zhou et al., 2011). Thorough *in vivo* studies could clarify the picture, but time and resources rarely allow it (Rostami-Hodjegan and Tucker, 2007). This is where artificial intelligence enters the conversation: it can forecast multi-target interactions and adverse effects early enough to cut down on extensive experimentation (Vamathevan et al., 2019). Computer-aided methods have helped bear the experimental load for years, but the real shift came when machine learning and deep learning began moving from the periphery to the center of pharmaceutical research (Lu et al., 2023). The growing stockpile of chemical and biological data, married to GPU-powered computation, opened the door to large-scale analyses that simply were not practical before (Rifaioglu et al., 2019). AI now tunes molecular design and property prediction in the early stages (Arnold, 2023) and later refines clinical trial design by spotting biomarkers and stratifying patients (Gupta et al., 2021). You find it everywhere: GNNs sniffing out protein interactions, GANs dreaming up new molecules, Transformers parsing patient records for smarter trials (Jiménez-Luna et al., 2021; Gangwal et al., 2024). Still, translating an AI-discovered candidate into an approved medicine remains early days. Two molecules capture the split reality: a fibrosis small molecule that advanced to Phase IIa with a positive efficacy signal, and a neuropsychiatric candidate that stopped in Phase I on safety grounds (Ren et al., 2025). Faster discovery does not automatically mean clinical success. These opposing outcomes illustrate AI’s power to compress timelines while exposing the gaps—particularly in safety prediction—that still need closing. As the algorithms evolve, they will continue to reshape the pipeline, but rigorous experimental validation is not going anywhere.

**FIGURE 1:**
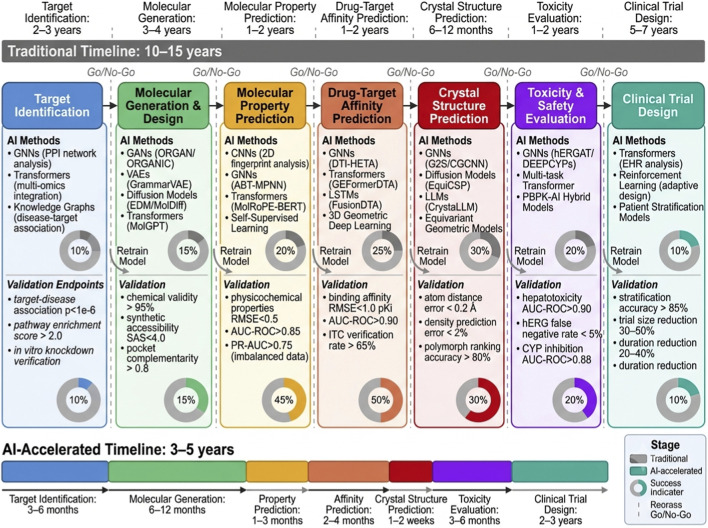
Integrated AI workflow across the drug discovery pipeline with traditional vs. AI-accelerated timelines.

Despite decades of refinement, conventional computer-aided drug design (CADD) approaches—quantitative structure-activity relationship (QSAR) modeling, molecular docking, and molecular dynamics simulations—have not substantially improved clinical success rates. These methods typically rely on static representations of protein–ligand interactions, overlook the dynamic nature of biological systems, and cannot fully capture polypharmacology, off-target effects, or the intricate interplay between pharmacokinetics and pharmacodynamics. Consequently, Phase II attrition due to lack of efficacy still hovers around 50%–60%, with toxicity causing another 30% of failures, figures that have barely changed over the past 2 decades (Sun et al., 2022). This persistent failure underscores the need for AI-driven strategies that integrate multimodal data, learn from complex patterns, and adapt across the entire drug development continuum. While several reviews have summarized individual AI tools, there is a lack of a comprehensive synthesis that connects foundational algorithms to clinical translation, including crystal structure prediction, safety assessment, and evolving regulatory frameworks. The present review therefore aims to fill these gaps by providing a systematic overview of AI methods spanning the full drug discovery pipeline, critically evaluating real-world case studies, and identifying key challenges in data, biology, and regulation that must be overcome to achieve clinical impact. Figure 1 illustrates how artificial intelligence integrates into each stage of the drug discovery pipeline, from target identification to clinical trials, contrasting traditional timelines with AI-accelerated workflows.

## 2 Review methodology

This systematic review was conducted in accordance with the Preferred Reporting Items for Systematic Reviews and Meta-Analyses (PRISMA) guidelines. A comprehensive literature search was performed in PubMed, Web of Science, Scopus, and Google Scholar for articles published between January 2007 and April 2026. The search strategy combined keywords and Boolean operators as follows: (“artificial intelligence” OR “machine learning” OR “deep learning”) AND (“drug discovery” OR “drug development” OR “target identification” OR “molecular property prediction” OR “crystal structure prediction” OR “drug-target interaction” OR “toxicity prediction” OR “generative models” OR “graph neural networks” OR “transformers” OR “diffusion models”).

Studies were included if they were peer-reviewed original research or review articles written in English and reported applications of AI or machine learning to any stage of drug discovery. Articles focusing solely on computational chemistry without biological or translational validation, as well as editorials, commentaries, and preprints, were excluded. After removal of duplicates using EndNote, two authors independently screened titles and abstracts, then retrieved and assessed full-text articles against the eligibility criteria. A total of approximately 1,850 records were initially identified; after deduplication, 1,200 records were screened at title/abstract level, 340 full-text articles were evaluated, and 96 studies met the inclusion criteria and were included in the final synthesis (Supplementary Figure S1). Disagreements were resolved by consensus. Owing to the heterogeneity of study designs—ranging from benchmark machine learning studies to clinical case reports—a formal risk-of-bias tool could not be applied uniformly. Instead, we qualitatively appraised study quality by considering data provenance, sample size, validation methodology (e.g., independent test sets, prospective validation), and transparency of reporting.

## 3 Artificial intelligence technology

### 3.1 Convolutional neural networks (CNNs)

Convolutional neural networks, a cornerstone of computer vision, excel at processing image-like data (Gu et al., 2015). Their key advantage is parameter sharing across filters, which reduces learnable parameters and speeds computation. In drug discovery, CNNs analyze bioactivity profiles from high-throughput microscopy and predict critical molecular properties, including ADMET profiles, activity, and toxicity (Vaz and Balaji, 2021; Rogers and Hahn, 2010). They also advance molecular characterization via circular fingerprints and predict molecular free energy from 2D structural representations, aiding stability and reactivity assessment. Recent extensions include 3D molecular structure analysis, such as parsing cryo-electron microscopy (cryo-EM) images of protein complexes. A 2024 Nature Methods study showed that CNNs can extract high-resolution features from cryo-EM density maps, accurately predicting protein-ligand binding conformations and outperforming traditional template-based methods in resolving flexible regions (Terashi et al., 2024). This application enhances structural biology-driven drug design by bridging low-resolution imaging and atomic-level modeling. While CNNs are highly effective for grid-structured data, they are inherently limited when applied to irregular graph structures; for molecular graphs, graph neural networks (GNNs) often yield superior accuracy, and for long-range sequential dependencies, Transformer architectures have become the preferred choice. Another notable pharmaceutical application is the use of three-dimensional convolutional networks to directly identify ligand-binding pockets from volumetric electron density maps, as implemented in the DeepSite predictor (Jiménez et al., 2017). These 3D-CNN approaches capture spatial context more comprehensively than two-dimensional methods but incur significantly higher computational costs, often necessitating GPU clusters for large-scale virtual screening. Consequently, in practice, CNNs are most efficiently deployed when combined with dimensionality reduction or transfer learning to mitigate resource demands.

### 3.2 Recurrent neural networks (RNNs)

Recurrent neural networks are designed for sequential data. They introduce cyclic connections within the same hidden layer, allowing the network to handle input sequences—a feature that makes them well suited to language processing. However, RNNs face challenges such as gradient explosion or vanishing due to long-term dependencies. In drug development, RNNs often represent molecules as strings (e.g., SMILES), providing new tools for molecular design and efficacy prediction (Bjerrum, 2018). Studies show that RNNs can predict chemical properties using features derived from SMILES representations, highlighting their potential in molecular descriptor learning. By capturing molecular structures and features effectively, RNNs can predict chemical and biological activity (Segler and Waller, 2017). Methods leveraging long short-term memory (LSTM) networks handle SMILES sequences for drug-target interaction prediction, enhancing accuracy and reliability. Despite these early successes, RNNs are being increasingly supplanted by Transformer-based models in sequence-driven drug discovery tasks. Transformers use parallelizable self-attention mechanisms that naturally capture long-range dependencies without the vanishing gradient limitations inherent in recurrent architectures, and they scale efficiently to longer sequences. A direct benchmark comparing LSTM and Transformer architectures for SMILES-based molecular generation found that the Transformer attained higher chemical validity, uniqueness, and training speed (Gupta et al., 2018). Nevertheless, RNNs retain value in domains where temporal order is paramount—such as pharmacokinetic time-series modeling or the analysis of continuous physiological signals from wearable sensors—where the inductive bias of recurrence aligns with the data. Therefore, while Transformers now dominate *de novo* molecular design, recurrent networks continue to serve niche but important roles in drug development.

### 3.3 Graph neural networks (GNNs)

Graph neural networks have grown in importance because they can abstract data into nodes and edges modeled as graphs. Molecular structures are naturally represented as graphs, with nodes for atoms and edges for chemical bonds. Integrating GNNs with drug discovery techniques accelerates the search for new drugs, improves candidate design, and enhances efficacy and safety (Ma and Lei, 2023). For example, SchNet is a GNN-based architecture specifically designed for predicting molecular properties and activities (Schütt et al., 2018). PotentialNet is another GNN approach for modeling and predicting potential energy surfaces between molecules, and DimeNet focuses on handling three-dimensional molecular structural data (Gasteiger et al., 2020). Each of these architectures comes with its own trade-offs. SchNet prioritizes computational speed by using continuous-filter convolutional layers that model interatomic interactions across varying distance scales, which makes it well suited for large-scale virtual screening. DimeNet sacrifices some of that speed to chase accuracy: it explicitly encodes directional information—bond angles, via Bessel function expansions and spherical harmonics—and delivers markedly better predictions for quantum mechanical properties, though at a substantially higher computational cost. PotentialNet, by contrast, is tuned specifically for potential energy surfaces. It captures both intramolecular and intermolecular interactions through a series of message-passing steps, which makes it particularly valuable for conformational analysis and binding affinity predictions.

### 3.4 Generative adversarial networks (GANs)

Generative adversarial networks consist of two components: a generator and a discriminator (Gürsakal et al., 2022). The generator creates new data points from random distributions, while the discriminator evaluates whether samples come from the training data or the generator. Training involves a min-max loss function, with both components optimizing adversarial objectives to achieve balance (Wang et al., 2024). This adversarial training improves the generator’s ability to produce realistic, conforming samples. GANs have achieved significant success in image generation, speech synthesis, and natural language processing, opening new possibilities for deep learning. In drug discovery, models like ORGAN and its derivative ORGANIC leverage GANs’ generative power, combined with domain-specific knowledge and chemical rules, to perform molecular generation—effectively producing new structures with desired properties (Ahmad et al., 2024; Pavan Kumar and Jayagopal, 2021). To ensure that GAN-generated molecules are chemically meaningful, a series of *post hoc* validity checks are routinely applied. These include enforcing valency and bond-order rules, canonicalizing SMILES strings with cheminformatics toolkits such as RDKit, and discarding any molecules that fail sanitization. Quantitative evaluation metrics commonly reported are the validity rate (the percentage of outputs that are chemically correct), uniqueness (the fraction of distinct structures within the generated set), and novelty (the proportion of generated molecules not present in the training set). These validation steps are essential to confirm that GAN outputs are not only drug-like but also potentially synthetically accessible.

### 3.5 Variational autoencoders (VAEs)

Variational autoencoders consist of an encoder and a decoder. Unlike conventional autoencoders, VAEs regularize and optimize their latent spaces (Nissen et al., 2021). During training, the encoder learns the latent structure of the data distribution, mapping inputs to mean and variance parameters, and samples latent variables (Z) from that distribution (Asperti et al., 2021). The decoder then reconstructs the input data from these variables. In drug discovery, VAEs generate novel molecular structures with specific chemical properties, accelerating the process and improving design efficiency and success rates (Salimi et al., 2024). Variants like GrammarVAE are specifically designed for molecular generation (Oliveira et al., 2022), while syntax-directed VAEs guide generation with syntactic rules, ensuring chemically valid molecules with desirable structural and functional properties (Griffiths and Hernández-Lobato, 2019). These models combine VAEs’ capabilities with domain knowledge to generate targeted molecular structures.

### 3.6 Diffusion models

Diffusion models generate data by gradually adding random noise and then learning to reverse the process to reconstruct samples (Ozbey et al., 2023). Their latent spaces are typically high-dimensional. In protein design, RF diffusion and Family-wide Hallucination leverage this framework: RF diffusion generates functional protein sequences by learning reverse diffusion from noisy inputs (Watson et al., 2023), while Family-wide Hallucination designs *de novo* luciferases with desired activity (Yeh et al., 2023). Recent advances include Equivariant Diffusion for Molecules (EDM), which enforces geometric constraints (e.g., bond angles, chirality) during molecular generation (Hoogeboom et al., 2023). EDM outperforms GANs and VAEs in producing chemically valid structures and preserves stereochemical properties critical for drug efficacy. Another breakthrough, MolDiff, integrates quantum mechanical descriptors into the diffusion process, enabling prediction of molecular conformations with accurate energy landscapes (Morehead and Cheng, 2024). These models address key limitations of earlier generative methods, such as unrealistic bond lengths or unstable conformations.

### 3.7 Transformer models

Transformer models consist of an input section, encoder, decoder, and output section, with the attention mechanism at their core. They replace recurrent connections with positional embeddings, enhancing the understanding of sequence order (Vaswani et al., 2017). The encoder converts input sequences into hidden representations, while the decoder generates target sequences; the attention mechanism enables global context integration. In drug discovery, Transformers have revolutionized protein structure prediction-for instance, AlphaFold2 uses a Transformer encoder to model residue-residue interactions with near-experimental accuracy (Jumper et al., 2021). Their parallel computing capability, unlike RNNs, allows efficient processing of long sequences (e.g., full-length proteins or multi-molecular complexes). For drug-target interaction prediction, Transformer-based models like ProtBERT (for protein sequences) and MolBERT (for SMILES) capture long-range dependencies. Transformers also optimize clinical trial design by analyzing electronic health records (EHRs) to identify patient subgroups, reducing trial size and duration. Table 1 summarizes the artificial intelligence methods discussed in this section, outlining their core mechanisms, primary tasks in drug discovery, and key validation endpoints.

**TABLE 1:** Summary of AI methods and their primary tasks in drug discovery.

AI method	Core mechanism	Primary tasks in drug discovery	Key validation endpoint
CNN	Local feature extraction via convolution	Bioactivity image analysis, ADMET prediction, cryo-EM density map parsing	Accuracy, AUC-ROC
RNN/LSTM	Sequential memory with gating	SMILES-based property prediction, DTI with sequences	RMSE, AUC-ROC
GNN	Message passing on molecular graphs	Affinity prediction (e.g., PotentialNet), molecular property modeling	RMSE, PR-AUC
GAN	Adversarial generator–discriminator	*De novo* molecular generation (ORGAN, ORGANIC)	Validity, uniqueness, novelty
VAE	Latent space regularization with encoder-decoder	Molecular generation (GrammarVAE), latent representation learning	Reconstruction accuracy, validity
Diffusion model	Reverse diffusion from noise	3D molecular conformation generation (EDM), protein design (RFdiffusion)	Chemical validity, stereochemical accuracy
Transformer	Self-attention with positional encoding	Protein structure prediction (AlphaFold2), DTI (ProtBERT, MolBERT), patient stratification	RMSD (Å), AUC-ROC, recall

## 4 Application of artificial intelligence technology in drug development

### 4.1 AI for molecular feature capture and extraction

In drug development, AI-driven machine learning models can automatically extract diverse data features after training on large datasets (Bhinder et al., 2021). Accurately describing drug molecules, proteins, and other large molecules is crucial, because it directly affects a model’s feature extraction capability (Cao et al., 2012). Researchers use various machine learning methods and molecular representations—from simple sequences to manually defined features—to describe these molecules effectively (Redkar et al., 2020). The choice of data representation significantly influences model pre-training and performance; a rational representation can markedly enhance predictive models (Rifaioglu et al., 2020). These efforts enable better understanding and use of chemical data, advancing drug development. Molecular fingerprints (FPs) are binary vectors indicating the presence or absence of specific substructures (Cereto-Massagué et al., 2015). Descriptors for substituent atoms, bonds, fragments, and functional groups reside in 1D, while atomic connectivity and molecular topology are in 2D. Current FPs face challenges such as dependence on large datasets for feature extraction and potential feature loss (Gao et al., 2020). Molecular graphs offer an intuitive representation with nodes (atoms) and edges (bonds), but storing the required matrices demands substantial disk space and memory, potentially reducing efficiency in molecular generation (David et al., 2020). SMILES sequences represent molecules as strings converted into one-hot vectors for machine learning. Compared with graphs, SMILES strings incur lower computational costs, but may lose structural information by not directly encoding atomic connectivity (Öztürk et al., 2016). Each representation has advantages and disadvantages; researchers must select suitable approaches based on task requirements and computational resources to enhance model performance and efficiency. To facilitate a clear, side-by-side comparison of these three dominant molecular representation paradigms, we summarize their core characteristics, trade-offs, and optimal use cases in Table 2. This comparative framework provides a practical guide for selecting the most appropriate representation method based on the specific goals and constraints of a given drug discovery task. For example, Ren et al. (2023) introduced SuHAN, a substructure hierarchical attention network that divides SMILES-formatted molecules into fragments and processes them through atomic and substructure layers to obtain multi-perspective features. Liu Y. et al. (2023) used rotary position embedding (RoPE) to encode SMILES sequences, enhancing BERT models’ ability to extract latent substructure information and improve molecular property prediction accuracy. Ji et al. (2022) proposed ReLMole, a molecular graph representation learning method that extracts complex structural features through layered graph modeling and a two-layer graph similarity contrastive learning scheme, offering new solutions for analyzing complex molecular structures.

**TABLE 2:** Comparative summary of molecular representation methods in AI-driven drug discovery.

Representation method	Core data structure	Computational complexity	Information retention	Key advantages	Primary limitations	Optimal use cases
Molecular fingerprints	Binary vector indicating presence/absence of substructures	Low	Low (loses 3D geometry and detailed connectivity)	- Extremely fast computation and comparison- well-established for large-scale virtual screening- low memory footprint	- Feature collision (different structures map to same fingerprint)- loses stereochemical and conformational information- limited interpretability	High-throughput virtual screeningSimilarity-based compound rankingRapid ADMET property predictionLibrary diversity analysis
SMILES sequences	Linear ASCII string encoding molecular topology	Medium	Medium (retains atomic connectivity but loses 3D geometry)	- Compact storage format- compatible with all NLP architectures (transformers, LSTMs)- easy data augmentation via canonicalization	- Ambiguous (one molecule can have multiple SMILES strings)- structural information is implicit rather than explicit- sensitive to minor sequence perturbations	*De novo* molecular generationSequence-based property predictionTransformer pre-training (e.g., ChemBERTa, MolFormer)Large-scale chemical database indexing
Molecular graphs	Nodes (atoms) connected by edges (chemical bonds)	High	High (retains atomic, bond, and topological information; extensible to 3D)	- Intuitive, chemically meaningful representation- naturally captures local and global structural features- supports integration of 3D conformational data	- High computational cost and memory usage- less mature for generative tasks compared to sequence models- requires specialized GNN architectures	Drug-target binding affinity prediction3D conformation generationHigh-accuracy molecular property modelingStructure-based drug design

### 4.2 AI for predicting drug-target binding affinity

Predicting drug-target interactions (DTI) is crucial for targeted therapy development (Vázquez et al., 2020). Deep learning models now dominate affinity prediction tasks, offering advantages in handling complex molecular data through layered learning and information propagation, thereby enhancing accuracy (Kong and Yu, 2018). Recent innovations have further improved DTI prediction. Wang et al. developed an LSTM-based model using position-specific scoring matrices and Laplacian matrices to extract protein evolutionary features, achieving an AUC-ROC of 0.89 after feature compression via sparse principal component analysis (Wang et al., 2020). Yuan et al. (2022) proposed FusionDTA, an LSTM architecture with multi-head linear attention that outperformed traditional max-pooling in global information aggregation, yielding an RMSE of 0.62 on the KIBA dataset. Shao et al.’s DTI-HETA (Shao et al., 2022), a GNN-GAT hybrid on heterogeneous graphs, achieved an AUC-ROC of 0.91 by highlighting key neighborhood node contributions. Notably, geometric Transformer-based models like GEFormerDTA integrate 3D molecular conformations and protein structure geometry via attention mechanisms, outperforming previous methods by capturing subtle steric effects and allosteric interactions (Liu et al., 2024). These advancements demonstrate a shift toward multi-modal feature integration in DTI prediction, bridging sequence-based and structural biology-driven approaches.

### 4.3 AI for crystal structure prediction

Crystal structure determines the three-dimensional periodic arrangement of molecules in a crystal lattice and critically influences pharmaceutical properties such as polymorphic stability, solubility, dissolution rate, and manufacturability (Gao et al., 2017). Polymorphism-the ability of a compound to exist in multiple crystal forms with distinct lattice energies and packing arrangements-poses both opportunities and risks (Desiraju, 2003). Different polymorphs can exhibit markedly different bioavailability and physicochemical profiles (Rohani, 2010). Traditional crystal structure prediction (CSP) methods rely on lattice energy minimization combined with global searches over possible packing arrangements, followed by density functional theory (DFT) calculations for energy ranking. While theoretically rigorous, this approach is computationally expensive and often fails to correctly rank polymorph stability at room temperature because of the subtle free-energy differences involved (Price, 2014). AI has advanced CSP by accelerating energy evaluation, structure ranking, and candidate generation. A landmark contribution came from Lemm et al., who developed Graph-To-Structure (G2S)-a graph neural network that directly predicts interatomic distances for out-of-sample molecules, transition states, and crystalline solids without explicit energy minimization (Lemm et al., 2021). G2S achieved mean absolute interatomic distance prediction errors below 0.2 Å with fewer than 8,000 training structures, matching or exceeding conventional structure generators while bypassing the accuracy-cost trade-off of force-field and *ab initio* methods. In parallel, Xie and Grossman introduced the Crystal Graph Convolutional Neural Network (CGCNN) framework, which directly learns material properties from the connectivity of atoms in the crystal, providing a universal and interpretable representation of crystalline materials (Xie and Grossman, 2018). Trained on over 10,000 crystals from the Materials Project database, CGCNN achieves DFT-level accuracy for eight different properties and enables extraction of atomic-level contributions to global properties, offering mechanistic insights into structure-property relationships.

Geometric deep learning has recently pushed molecular CSP forward. Kilgour et al. developed two complementary models (Kilgour et al., 2023a): MolXtalNet-D predicts crystal density with a mean absolute error below 2% on diverse test sets, and MolXtalNet-S ranks crystal stability, reliably distinguishing experimental structures from synthetic decoys when validated on Cambridge Structural Database Blind Tests. Both are computationally cheap enough to drop into existing CSP pipelines for search-space reduction and candidate scoring (Kilgour et al., 2023b). At the same time, generative models are carving out their own role. A 2024 study presented Cond-CDVAE, a conditional crystal diffusion variational autoencoder trained on over 670,000 structures (including high-pressure phases from the Materials Project database). It predicted a high percentage of unseen ambient-pressure experimental structures, matching or beating conventional global optimization methods (Luo et al., 2024). CrystaLLM tackles the problem from an entirely different angle, reframing crystal structure generation as an autoregressive language modeling task on CIF files; trained on millions of CIFs, it produces plausible structures for inorganic compounds never seen during training (Antunes et al., 2024). Equivariant diffusion models have also advanced: EquiCSP rigorously maintains lattice permutation, rotation, and periodic translation symmetries throughout training and inference, significantly outperforming earlier models in both accuracy and convergence speed. For molecular crystals, diffusion models have been extended to predict packing arrangements by incorporating periodic boundary conditions and symmetry constraints. Despite this progress, most AI-based CSP models still hunger for large, high-quality training sets of experimentally determined structures—resources that are scarce for complex drug molecules. Predicting kinetic (metastable) polymorphs, which routinely appear in industrial crystallization because of nucleation kinetics and processing conditions, remains an open problem; nearly all models fixate on thermodynamic stability ranking. Future efforts will likely combine generative AI with physics-based refinement, embed CSP within end-to-end formulation design, and develop models capable of predicting both stable and metastable forms under processing-relevant conditions.

### 4.4 AI for predicting molecular properties

Advances in machine and deep learning have made molecular property prediction an increasingly important yet challenging task in drug discovery (Li Z. et al., 2022). Compound properties fall into three main classes: physicochemical, biophysical, and physiological, each with benchmark datasets (Wu et al., 2018). Model performance is typically assessed via classification and regression tasks, using AUC-ROC to evaluate accuracy and robustness and RMSE to measure prediction error magnitude (Wallström and Segerstedt, 2010). It is important to note that AUC-ROC can be overly optimistic in highly imbalanced biomedical datasets, where negative samples greatly outnumber positive ones—a common scenario in virtual screening for rare toxicities or low-frequency off-target effects. In such cases, the precision-recall AUC (PR-AUC) or F1-score provides a more informative assessment of model performance. Recent guidelines for AI in drug discovery recommend reporting both ROC-AUC and PR-AUC. For sparse screening data, we encourage researchers to adopt PR-AUC as the primary metric, supplemented by F1-score at optimized classification thresholds. Enhancing data augmentation methods based on SMILES sequences is significant for molecular property prediction. Jiang et al. (2023) introduced NoiseMol, which injects perturbative noise into atom-level or substructure-level labeled SMILES strings to systematically expand dataset size, alleviating labeled data constraints and demonstrating improved predictive capability and robustness of Transformer models. Li C. et al. (2022) proposed the Multiple SMILES method, generating multiple SMILES encodings per molecule as an automated data augmentation strategy that mitigates overfitting from insufficient data. According to Liu C. et al. (2023), ABT-MPNN—a variant of graph convolutional networks (GCNs) that integrates end-to-end representations at bond, atom, and molecule levels—outperforms benchmark models across various datasets, particularly in quantitative structure-property relationship evaluation.

Biomedical datasets are often small, imbalanced, or lacking labeled examples—particularly for rare diseases, novel targets, or proprietary compounds. Three complementary machine learning paradigms address these limitations. Few-shot learning (FSL) enables models to generalize from very few labeled examples (e.g., 1–10 compounds) by learning metric spaces or meta-learning strategies. In drug discovery, prototype-based FSL has been applied to predict toxicity for structurally novel compounds where only a handful of analogues are available. Self-supervised learning (SSL) pre-trains models on large, unlabeled chemical or protein databases (e.g., 1.5 billion SMILES strings from PubChem, or 200 million protein sequences from UniRef) using pretext tasks such as masked language modeling or contrastive learning. SSL-trained encoders (e.g., MolCLR for molecules, ESM-2 for proteins) capture rich structural and functional semantics, then require only fine-tuning on small labeled datasets—dramatically reducing the need for expensive annotations. Transfer learning extends this paradigm by taking a model pre-trained on a large, related task (e.g., general molecular property prediction on ChEMBL) and adapting it to a target task with limited data (e.g., predicting binding affinity for a specific kinase). Transformer-based models like ChemBERTa and MolFormer exemplify this approach. Collectively, these methods mitigate data scarcity, lower the barrier for rare-disease drug development, and reduce reliance on proprietary datasets.

### 4.5 AI-driven molecular generation and optimization

AI has become a cornerstone in the generative phase of drug discovery, addressing the dual challenges of exploring vast chemical spaces and designing molecules with tailored properties. A foundational task is generating biologically relevant, low-energy molecular conformations, critical for accurate binding mode prediction and property assessment. Traditional methods rely on conformational search coupled with energy minimization techniques such as molecular mechanics or DFT (Hawkins, 2017); deep learning offers data-driven alternatives that greatly accelerate the process. Early approaches, such as the VAE framework by Mansimov et al. (2019) for direct coordinate generation, faced challenges in maintaining roto-translational invariance. Subsequent models improved robustness using intermediate representations; for instance, Xu et al. (2021) employed flow models to generate distance matrices refined via distance geometry and dual-layer optimization, while Zhu et al. (2022) demonstrated the efficacy of directly predicting atomic coordinates. The field has been further advanced by diffusion models, which enforce geometric constraints during a learned denoising process to yield chemically valid and stereochemically accurate conformations with high success rates. Beyond conformation, *de novo* molecular design leverages AI to generate novel, synthetically accessible compounds. Sequence-based models, inspired by natural language processing, treat molecules as strings (e.g., SMILES). LSTM networks have generated novel scaffolds for specific targets like p300/CBP inhibitors, and Transformer-based architectures like MolGPT enable controlled generation conditioned on desired properties (Yang et al., 2020; Bagal et al., 2022). Concurrently, graph-based methods provide a more intuitive representation, with models such as the Graph Convolutional Policy Network (GCPN) combining GNNs with reinforcement learning for goal-directed generation, and graph VAEs like NeVAE handling variable-sized molecular graphs. To capture complex interactions, architectures like the Multi-Physics Graph Neural Network (MP-GNN) integrate multi-scale features. The ultimate objective is target-oriented molecular optimization, where AI integrates generative and predictive models into a closed-loop system. In this cycle, generators propose candidates evaluated by predictors for key properties (e.g., affinity, solubility), with reinforcement learning often providing the optimization policy. This approach allows conditioning generation not only on simple properties but also on the 3D geometry of target binding pockets, leading to molecules designed for geometric and chemical complementarity. This integrated AI-driven pipeline-from conformation sampling to targeted *de novo* design and iterative optimization-streamlines hit-to-lead and lead optimization phases.

### 4.6 Real-world case studies

**FIGURE 2:**
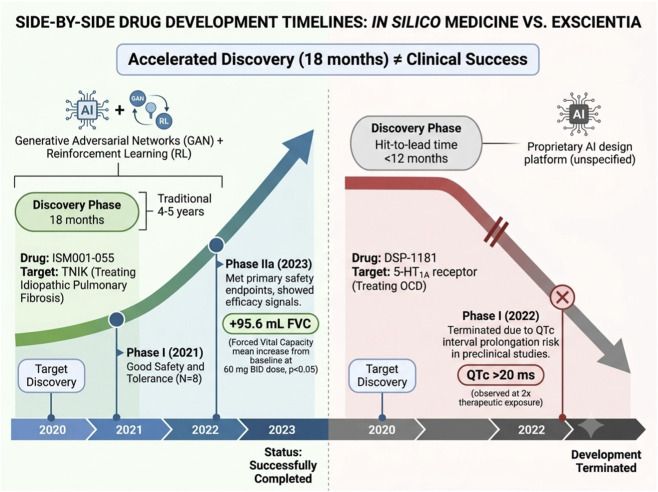
Comparative clinical development timelines of two AI discovered small molecules with distinct clinical outcomes.

Recent integration of AI into drug discovery has produced both notable successes and instructive failures. A balanced examination of real-world cases provides critical insights into AI’s current capabilities and limitations. ISM001-055, a small-molecule inhibitor of TNIK (traf2-and NCK-interacting kinase) for idiopathic pulmonary fibrosis (IPF), was developed by Insilico Medicine using a generative adversarial network combined with reinforcement learning (Ren et al., 2025; Aladinskiy et al., 2024). The AI platform identified TNIK as a novel target by analyzing multi-omics data from IPF patient tissues, then generated and optimized candidate compounds *in silico*. The entire preclinical discovery phase-from target identification to lead optimization-took approximately 18 months, compared with the typical 4–5 years. In 2021, ISM001-055 entered a Phase I clinical trial, which demonstrated favorable safety and tolerability. A subsequent Phase IIa trial (completed in 2023) met its primary safety endpoints and showed dose-dependent improvements in forced vital capacity (FVC), a key efficacy measure in IPF (Xu et al., 2025). These results, reported at the 2023 American Thoracic Society International Conference, represent the first prospective validation of an AI-discovered and AI-designed drug reaching Phase II with positive efficacy signals. DSP-1181, a long-acting 5-HT_1_A receptor agonist for obsessive-compulsive disorder, was discovered through a collaboration between Exscientia and Sumitomo Pharma. AI-driven design reduced the hit-to-lead timeline to under 12 months, demonstrating AI’s ability to accelerate early-stage chemistry (Paul and Mital et al., 2025). However, in 2022, the compound was discontinued during Phase I after preclinical safety studies revealed a prolonged QT interval in animal models-a known risk factor for cardiac arrhythmia. This case illustrates that accelerated hit identification does not guarantee clinical progression and underscores the need for better integration of safety and pharmacokinetic prediction into AI-driven design pipelines. Figure 2 contrasts the development timelines and outcomes of two representative AI-discovered drugs, highlighting both the accelerated discovery phase and the remaining challenges in safety prediction that led to different clinical fates. Table 3 provides additional real-world examples of AI-discovered compounds that have progressed to clinical development, illustrating the diversity of AI methods, accelerated timelines, and clinical outcomes.

**TABLE 3:** Real-world case studies of AI-discovered drugs in clinical development.

Compound	Company	AI method	Target/Indication	Timeline reduction (discovery to lead)	Clinical status (as of 2025)
ISM001-055	Insilico medicine	GAN + reinforcement learning	TNIK/Idiopathic pulmonary fibrosis	∼18 months (vs. typical 4–5 years)	Phase IIa completed (positive efficacy)
DSP-1181	Exscientia + sumitomo pharma	Generative AI (proprietary)	5-HT_1_A receptor/OCD	<12 months (hit-to-lead)	Discontinued in phase I (QT prolongation in preclinical)
REC-994	Recursion (now exscientia)	CNN on high-content imaging	Superoxide/Cerebral cavernous malformation	3 years (vs. typical 5–6)	Phase II ongoing
AI-accelerated lead optimization (meta-analysis)	Various (20 programs)	GNN, transformer, RL	Multiple oncology and rare disease targets	30%–50% screening time reduction	Phase I/II attrition comparable to industry (safety failures predominant)

Beyond individual molecules, retrospective analyses of AI-native biotech companies have provided quantitative insights. A 2024 systematic review examined 20 AI-driven drug discovery programs from 2018 to 2023. It found that AI-assisted lead optimization reduced candidate screening time by 30%–50% on average, but the attrition rate in Phase I/II remained comparable to industry benchmarks, with safety issues and unexpected pharmacokinetic properties being the primary causes of failure (Jayatunga et al., 2024). This suggests that while AI excels at accelerating early-stage chemistry, its ability to predict complex *in vivo* behavior-especially toxicity and human-specific metabolism-remains an area for improvement. Large pharmaceutical companies have also reported internal metrics. AI-driven lead screening increased hit rates by five-fold for oncology targets, reducing the number of candidates entering preclinical testing by 40% while maintaining efficacy. Empirical internal data from leading pharmaceutical firms further corroborate the practical value of AI-enabled early toxicity filtering for lead prioritization. These real-world cases collectively demonstrate that AI can meaningfully accelerate and improve early-stage drug discovery, but clinical translation still requires rigorous experimental validation, iterative model refinement, and-crucially-transparent reporting of both successes and failures to guide future development.

### 4.7 AI for toxicity and safety evaluation

Toxicity prediction and safety assessment are critical for advancing compounds from discovery to clinical trials. Traditional *in vitro* and *in vivo* experiments are costly, time-consuming, and raise ethical concerns. AI models trained on public databases-such as Tox21, SIDER, and Open TG-GATEs-enable early detection of hepatotoxicity, cardiotoxicity, genotoxicity, and other adverse effects (Zhou et al., 2024). A key advancement is the prediction of off-target interactions that underlie many safety liabilities. Deep learning models integrating protein sequence homology, binding pocket structures, and ligand similarity have been developed. When evaluated on test sets of kinase inhibitors, these models reduce false negatives compared with traditional docking methods in identifying unintended hERG channel interactions (a major cause of QT prolongation and sudden cardiac death). They also predict off-target inhibition of cytochrome P450 enzymes (CYP2C9, CYP3A4) with high accuracy, enabling early screening of drug-drug interaction risks (Liu et al., 2025; Liu et al., 2023; Ai et al., 2023; Schifferstein et al., 2024). For cardiotoxicity, models using a combination of molecular fingerprints and graph neural networks predict hERG blockade and QT prolongation. Trained on thousands of compounds from the ChEMBL and PubChem bioassay databases, these models achieve AUC-ROC values above 0.90 on independent test sets, outperforming traditional QSAR models. Pharmaceutical companies have adopted that integrating these models into their early screening cascade excluded 30%–40% of high-risk compounds before synthesis, reducing the need for patch-clamp electrophysiology by half (Tran-Nguyen et al., 2025; Lee and Yoo et al., 2025; Mendez et al., 2019). AI also facilitates in vitro–in vivo extrapolation (IVIVE) by combining high-throughput screening data with physiologically based pharmacokinetic (PBPK) models. Multi-task deep learning models trained on the Open TG-GATEs database predict drug-induced liver injury (DILI) by integrating transcriptomic signatures from rat liver with human hepatocyte data, achieving significantly higher accuracy than models using either species alone. These examples demonstrate that AI can serve as an effective filter for safety liabilities, though regulatory acceptance still requires prospective validation and explainability (Chandra et al., 2025; Igarashi et al., 2015; O’Donovan et al., 2020; Zhang et al., 2025).

## 5 Challenges and prospects

### 5.1 Biological complexity and incomplete indices

Biological systems are messy, and the indices we use to describe them remain incomplete—a reality that puts hard limits on what AI can currently achieve. The stubbornly high attrition in the clinic is a shared bottleneck for AI-derived candidates, too. Unlike chemical data, which are relatively stable and computationally tractable, biological data are dynamic, context-dependent, and frequently non-linear. Consider a few concrete manifestations. Drug–microenvironment interactions—tumor pH gradients, extracellular matrix composition, immune cell infiltration—alter efficacy and resistance, yet most AI models lean on static chemical features such as molecular weight, lipophilicity, and polar surface area, ignoring these spatiotemporal dynamics. Gut microbiota-mediated metabolism can transform a parent drug into active, inactive, or toxic metabolites; irinotecan-induced severe diarrhea driven by microbial β-glucuronidase is a classic example, and neither standard *in vitro* assays nor most AI models capture it. Then there is concentration-dependent polypharmacology: a drug may hit different targets at different doses, producing divergent therapeutic and adverse effects, but many models simply assume a single dominant target based on literature or docking scores, missing the dose-dependent switching entirely.

To address these gaps, multi-scale AI modeling is emerging. This approach integrates molecular dynamics simulations (for protein–ligand binding kinetics) with systems biology networks (for pathway crosstalk) and agent-based models (for tissue-level heterogeneity). For example, a hybrid framework combining a graph neural network to predict binding affinities across multiple potential targets with an agent-based model of the tumor microenvironment has been shown to predict clinical responses with higher accuracy than ligand-based models alone and correctly identify resistance mechanisms missed by conventional affinity-only predictions. Another promising direction is the integration of single-cell multi-omics data: variational autoencoders have been used to map drug-induced transcriptional changes in thousands of individual cells, predicting hepatotoxicity with high accuracy and identifying previously unknown inflammatory pathways. To ensure the reliability of these multi-scale models, experimental validation strategies are being actively developed. Prospective validation loops, in which model predictions guide *in vitro* or *in vivo* experiments and the results are fed back to refine the models, are becoming increasingly important. Organ-on-chip platforms that recapitulate key aspects of the tumor microenvironment, for instance, have been employed to test AI-predicted drug responses under controlled, human-relevant conditions, enabling iterative improvement of both the computational and the biological models. In the field of neurodegeneration, researchers have combined molecular dynamics simulations of tau protein misfolding with neuronal network models to predict the spatiotemporal spread of tau pathology in Alzheimer’s disease. These hybrid models are being validated against positron emission tomography imaging and cerebrospinal fluid biomarkers, offering a promising path toward AI-driven prediction of disease progression and treatment response. Such integrated validation frameworks will be essential to move multi-scale AI models from proof-of-concept to regulatory-grade decision-making tools. Despite these advances, major challenges persist. Most multi-scale models require extensive parameterization and computational resources, limiting scalability. Moreover, biological indices used as model inputs (e.g., gene expression levels, protein concentrations) are often measured with high noise and batch effects. Standardizing data collection protocols and developing robust noise-aware AI architectures (e.g., Bayesian neural networks) are essential next steps. Without better representation of biological dynamics, AI predictions will remain incomplete for complex human diseases such as cancer, neurodegeneration, and autoimmune disorders.

### 5.2 Data quality and talent shortages

AI development in drug discovery faces universal challenges in data quality and interdisciplinary talent availability. Globally, pharmaceutical big data suffer from insufficient volume for rare diseases, fragmented storage across proprietary databases, and inconsistent standards for metadata annotation. Critical data types—such as negative experimental results, real-world adverse event reports, and detailed pharmacokinetic data from failed clinical trials—are often inaccessible due to commercial confidentiality or publication bias. Even when data are shared, lack of standardization (e.g., variations in clinical coding systems such as ICD-10 vs. SNOMED CT, or differences in chemical representation formats) limits cross-institutional model training. Mitigating these data challenges requires global adoption of FAIR (Findable, Accessible, Interoperable, Reusable) principles and common standards such as CDISC (Clinical Data Interchange Standards Consortium) for clinical data, SMILES for chemical structures, and PDBx/mmCIF for macromolecular structures; the FAIR framework specifies unified criteria for data archiving and open sharing to break data silos across academia and pharmaceutical enterprises. Initiatives like the European Union’s IMI (Innovative Medicines Initiative) and the US NIH’s Bridge2AI program exemplify efforts to build standardized, AI-ready datasets. In parallel, the talent gap stems from the need for expertise spanning deep learning architectures, medicinal chemistry, pharmacokinetics, and regulatory science. While specialized training programs have emerged—such as the “AI in Drug Discovery” fellowships at MIT and Cambridge, and AI-pharmacy joint degrees in China and South Korea—the demand far exceeds supply. Addressing this shortage requires fostering industry-academia partnerships, continuous education via workshops and online courses, and integration of AI literacy into pharmaceutical curricula worldwide.

### 5.3 Multimodal data integration and explainable AI

Relying on single-modal data (e.g., molecular structures or gene expression) fails to capture the full complexity of drug-target interactions and biological systems. Multimodal data integration-combining genomics, proteomics, metabolomics, clinical imaging, and real-world evidence-offers a more comprehensive view of disease mechanisms and drug effects. For example, integrating single-cell RNA sequencing (scRNA-seq) data with molecular docking results via graph attention networks (GATs) can reveal how drug-induced transcriptional changes in specific cell subtypes correlate with binding affinity, enhancing *in vivo* efficacy predictions. Technical hurdles include heterogeneous data formats (e.g., 3D protein structures vs. tabular clinical data) and noise. Advanced AI techniques address this: multi-task learning models jointly train on molecular property prediction and patient response data, leveraging shared features to improve performance; adversarial training reduces batch effects between datasets, ensuring consistent biological signal representation. Explainable AI (XAI) is critical for transparency, regulatory approval, and trust. Core XAI techniques include LIME (Local Interpretable Model-Agnostic Explanations) and SHAP (SHapley Additive exPlanations): LIME locally disturbs input features to quantify single-variable prediction contribution, whereas SHAP calculates feature importance based on cooperative game theory, both of which identify key molecular features (e.g., specific functional groups) or biological pathways driving predictions. For instance, applying SHAP to a GNN-based toxicity model revealed that a benzene ring substitution pattern strongly correlated with hepatotoxicity, guiding medicinal chemists to modify lead compounds.

**FIGURE 3:**
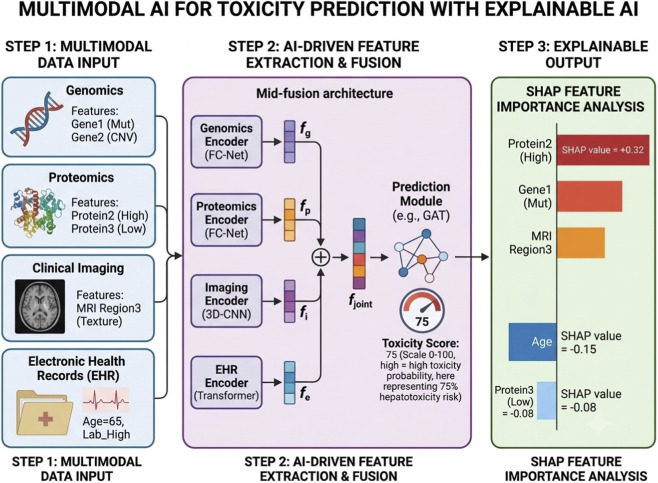
Multimodal AI-driven toxicity prediction model and explainable feature importance analysis.

While SHAP, LIME, and attention-based methods provide valuable transparency, it is critical to recognize that they offer *post hoc* interpretability rather than true causal inference. These methods approximate model behavior locally but do not guarantee that the highlighted features correspond to underlying biological mechanisms. For example, a SHAP analysis might identify a particular functional group as ‘important’ for hepatotoxicity prediction based on statistical correlations in the training data, yet that group might be a spurious correlate of a true toxicophore or might reflect batch effects in assay data. Moreover, *post hoc* explanations can be inconsistent across different XAI methods applied to the same model, and they are sensitive to input perturbations and hyperparameter choices. Recent work has proposed causal XAI frameworks that integrate structural causal models or counterfactual reasoning to distinguish correlation from causation, but these remain computationally intensive and require prior knowledge of causal graphs. Therefore, while XAI is essential for regulatory acceptance and debugging, conclusions drawn from SHAP/LIME should be validated experimentally, and researchers should avoid overinterpreting feature importance as biological ‘proof’. Figure 3 shows how multimodal data sources–genomics, proteomics, clinical imaging, and electronic health records–are integrated via AI models, and how explainable AI techniques such as SHAP identify the key features driving predictions, thereby enhancing transparency and regulatory acceptance. Regulatory bodies like the FDA now encourage XAI integration in drug submissions, with recent guidelines recommending model interpretability reports for AI-driven efficacy predictions. Future directions include dynamic XAI, tracking how model reasoning evolves with new data (e.g., updated clinical trial results) to ensure ongoing reliability.

### 5.4 AI in Traditional Chinese Medicine and natural products

AI-driven drug discovery extends to Traditional Chinese Medicine (TCM) and natural products, addressing their unique challenges of multi-component systems and complex therapeutic mechanisms. TCM, with millennia of empirical data, involves herbal formulas where “monarch-minister-assistant-guide” interactions determine efficacy—AI helps decode these by analyzing herbal chemical fingerprints alongside clinical outcomes. For example, a study in the Chinese Journal of Natural Medicines used knowledge graphs to map connections between thousands of TCM formulas, identifying that combinations of Astragalus membranaceus (monarch) and Angelica sinensis (minister) consistently enhance immunomodulatory effects via synergistic regulation of IL-6 and TNF-α pathways. In natural product research, AI accelerates screening of plant-derived compounds. Generative models like TCM-GAN generate novel natural product-like structures by learning from phytochemical databases (e.g., TCMSP), ensuring synthetic feasibility while optimizing for desired properties (e.g., anti-inflammatory activity). Beyond mere generation, AI-guided optimization of existing natural products has yielded tangible clinical candidates. For instance, machine learning models applied to artemisinin derivatives have led to analogues with improved solubility and bioavailability, at least one of which has advanced to Phase I clinical evaluation. Similarly, a graph neural network screen of berberine analogues identified a compound with enhanced kinase inhibition and reduced off-target effects, now in late-stage preclinical development. AI has also expedited the isolation of bioactive molecules: deep learning-assisted fractionation recently accelerated the discovery of analgesic diterpenes from Euphorbia species, demonstrating how AI can streamline the typically laborious purification process. AI also aids safety assessments: graph neural networks have been applied to predict hepatotoxicity of TCM extracts, integrating metabolomic data to identify toxic metabolites formed during decoction—reducing false negatives compared with traditional *in vitro* assays. Challenges include standardizing TCM data (e.g., variable herbal processing methods) and improving model interpretability for complex formulas. Solutions involve establishing centralized databases (e.g., the China TCM Formula Database) with standardized metadata on cultivation and processing parameters, and developing TCM-specific XAI tools—such as herbal ingredient contribution scores—to clarify how individual herbs influence predictions. As these advances mature, AI will facilitate TCM’s global acceptance by bridging empirical knowledge with evidence-based validation.

### 5.5 Ethical, privacy, and regulatory considerations in AI-Driven drug discovery

AI integration into drug discovery introduces significant ethical, privacy, and regulatory challenges that must be addressed for responsible translation. Data privacy and security are paramount when using sensitive patient genomic and clinical data, and compliance with regulations like GDPR and HIPAA is essential; privacy-enhancing technologies such as federated learning provide feasible solutions to confidential data utilization. Algorithmic bias and fairness pose serious risks, as models trained on non-representative datasets can perpetuate health disparities by yielding skewed efficacy or safety predictions across diverse populations, which requires diversified dataset construction and bias-aware validation to mitigate adverse impacts. Accountability and explainability are critical for clinical trust and regulatory approval. The “black-box” nature of complex deep learning models necessitates the XAI tools including SHAP and LIME elaborated in Section 5.3 to clarify decision-making logic and establish clear responsibility frameworks for model prediction errors. Finally, global regulatory frameworks (FDA, EMA, NMPA) need continuous revision to accommodate adaptive AI tools, including developing dedicated approval pathways for continuously learning algorithms, establishing standardized model lifecycle management rules, and promoting international regulatory harmonization to balance technological innovation and patient safety. Proactive governance of the above issues is fundamental to the ethical and sustainable advancement of AI-driven drug development.

The FDA, EMA, and NMPA have formulated differentiated regulatory policies targeting AI in drug discovery. The FDA has taken the most proactive stance: it issued draft guidance on Artificial Intelligence and Machine Learning in Drug Development in 2023 and built an AI Steering Committee affiliated with the Center for Drug Evaluation and Research (CDER). The agency promotes model-informed drug development (MIDD) and accepts voluntary submissions of AI model documents through the MIDD pilot program. The EMA focuses on algorithmic transparency and published the Guideline on the use of artificial intelligence in the medicinal product lifecycle in 2024, which requires detailed documentation of AI development, validation and post-market monitoring and stresses mandatory human oversight for adaptive AI applications. China’s NMPA released the Guiding Principles for the Evaluation of Artificial Intelligence Medical Products in 2022 and launched drug-discovery-specific supplementary updates in 2025, with core evaluation criteria focusing on data provenance, algorithm stability and clinical validation based on domestic patient cohorts.

All three regulators face common unsolved challenges: establishing universal verification standards for continuously self-updating algorithms, designing reasonable model revision rules to avoid repetitive full re-approval after version iteration, and facilitating cross-border regulation alignment. The International Council for Harmonisation (ICH) is compiling a global reflection paper on AI in drug development (expected in 2027), which is anticipated to unify core regulatory requirements worldwide.

### 5.6 Reproducibility, version control, and model drift

Reproducibility remains a significant hurdle in AI-driven drug discovery. Subtle changes in training-test splits, random seeds, or software library versions can lead to substantially different model outputs. To address this, we recommend adopting model cards—standardized documentation that reports model architecture, training data provenance, hyperparameters, intended use cases, and performance on predefined benchmark sets. Model cards should be made publicly available alongside code and trained weights whenever possible. Second, data snapshots are essential: raw and processed datasets should be versioned (e.g., using Git LFS or Zenodo) to ensure that results can be precisely reproduced as datasets evolve over time. Third, the community should develop and maintain open benchmarks with fixed training/validation/test splits and standardized evaluation pipelines, similar to MoleculeNet or Therapeutics Data Commons, to enable fair comparisons across studies.

Another growing concern is model drift—the gradual degradation of predictive performance as new chemical and biological data become available or as target distributions shift (e.g., novel compound scaffolds, different assay conditions). In the context of continuous learning, where models are updated incrementally without full retraining, version control becomes even more critical. Without careful management, continuous learning can lead to catastrophic forgetting or unintended bias accumulation. Practical mitigation strategies include: (i) periodic recalibration on held-out test sets, (ii) using statistical process control charts to monitor prediction error over time, and (iii) maintaining a shadow ensemble that compares the current model against a frozen baseline. Regulatory agencies are beginning to require lifecycle management plans for AI models used in drug development, including version tracking and drift monitoring protocols.

## 6 Conclusion

AI has fundamentally changed the rhythm of drug discovery. Data-driven approaches—graph neural networks, diffusion models, Transformers—have shifted the paradigm from trial-and-error to rational, efficient optimization. The Phase IIa progression of an AI-designed TNIK inhibitor shows what compressed timelines can look like, but clinical failures driven by safety and *in vivo* unpredictability make it clear that substantial predictive gaps persist. Biological complexity, fragmented data, and a shortage of cross-disciplinary expertise continue to demand attention. Multimodal data fusion and explainable AI are slowly strengthening model transparency and regulatory acceptance, while the extension into Traditional Chinese Medicine and natural products widens the search for bioactive molecules. Looking ahead, foundation models, self-driving laboratories, and quantum-accelerated simulations hold the promise of even shorter development cycles and genuinely personalized therapies. Yet rigorous experimental validation and adaptive regulatory frameworks will remain non-negotiable. The convergence of AI and pharmaceutical sciences will ultimately deliver more precise, accessible treatments—provided that computational innovation keeps pace with robust biological and clinical evidence.
