# Supplementary figures and images for: Artificial intelligence in drug discovery: from algorithmic foundations to clinical translation
Source: Front Pharmacol. 2026 Jul 3;17:1870527. doi: 10.3389/fphar.2026.1870527 (PMC13375493; doi:10.3389/fphar.2026.1870527)

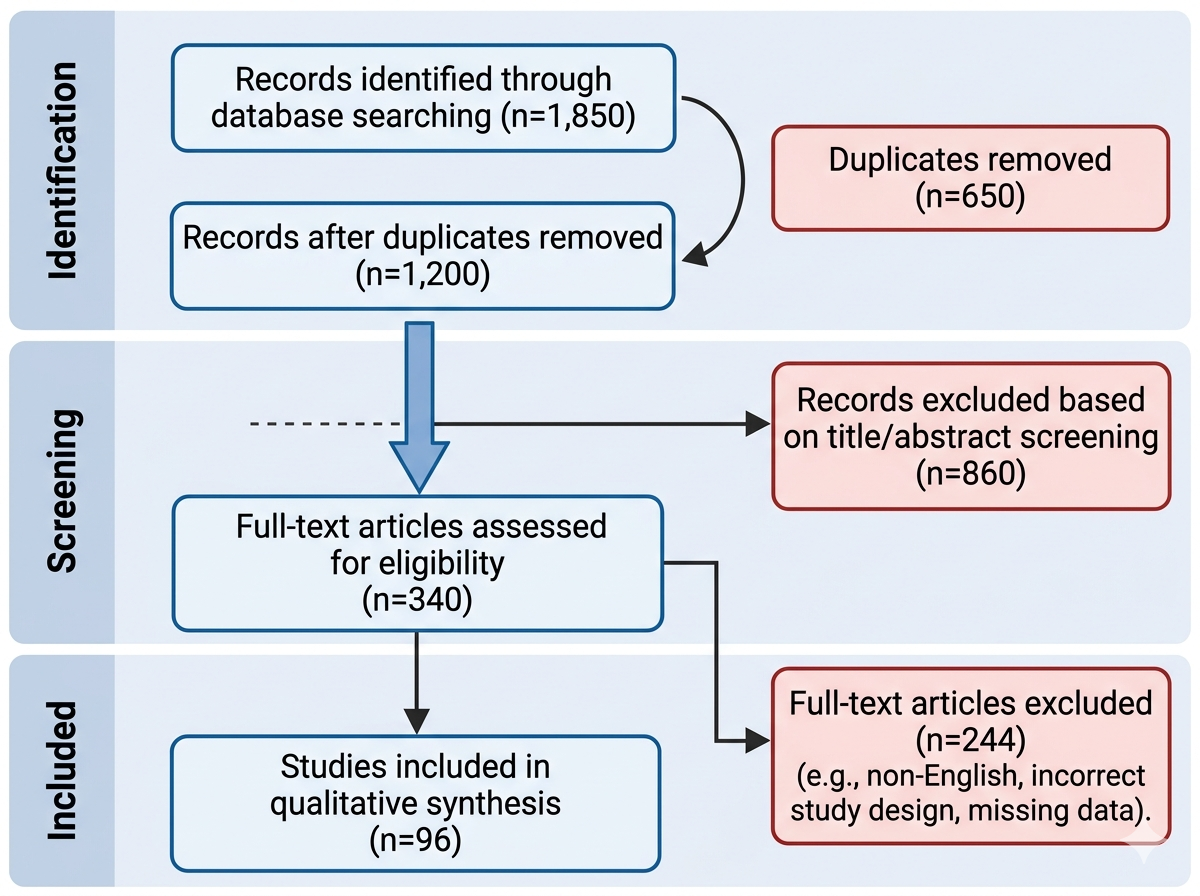

Supplement: Supplementary file 1 [file Image1.tif]
